# Ischemic Colitis Secondary to Ileocolic Artery Thrombosis in the Context of Active Ulcerative Colitis During Pregnancy: A Case Report and Review of the Literature

**DOI:** 10.7759/cureus.97001

**Published:** 2025-11-16

**Authors:** Dibanhy I Moreno-Mendoza, Jose A Vergara-Torrente, Alondra Esparza-González, Gerardo E Muñoz-Maldonado

**Affiliations:** 1 General Surgery, Hospital Universitario "Dr. José Eleuterio González" UANL, Monterrey, MEX

**Keywords:** arterial thrombosis, inflammatory bowel disease, ischemic colitis, severe truelove and witts, ulcerative colitis

## Abstract

Ulcerative colitis (UC) during pregnancy poses diagnostic and therapeutic challenges due to symptom overlap with normal pregnancy physiology and the maternal-fetal risks associated with active inflammation.

We present the case of a 24-year-old woman with a prior diagnosis of UC who discontinued treatment after finding out she was pregnant. At 22 weeks of gestation, she began experiencing abdominal pain and bloody diarrhea. Magnetic resonance imaging revealed thickening of the terminal ileum, ascending and transverse colon, with microhemorrhages, along with severe anemia and hemodynamic instability. The patient’s condition worsened, presenting signs of peritoneal irritation. An urgent laparotomy was performed, including partial ileectomy, right colectomy, and end ileostomy. Twelve hours after the procedure, intrauterine fetal demise was confirmed. Histopathological examination revealed ischemic colitis with Ileocolic arterial thrombosis, a rare complication of the disease that carries a high morbidity and mortality rate, and is scarcely documented in the medical literature.

Although no medical therapy completely eliminates the risk of flare-ups and complications, good adherence to treatment has been shown to achieve complication rates similar to those of non-pregnant women. This case highlights the importance of strict management of Inflammatory bowel disease during pregnancy and the need to avoid discontinuing maintenance therapy due to unfounded concerns about teratogenicity.

## Introduction

Ulcerative colitis (UC) is a type of inflammatory bowel disease (IBD) with multifactorial origins and uncertain etiology [1], and it is associated with multiple extraintestinal complications. Approximately 20% of patients with UC experience at least one severe exacerbation during the course of their disease [2]. Thromboembolic complications, although infrequent, are associated with high morbidity and mortality.

We present the case of a pregnant patient with a prior diagnosis of UC who experienced a severe flare according to the Truelove and Witts classification. During her clinical course, unusual complications were documented, including ischemic colitis secondary to arterial thrombosis and fetal loss. While both venous and arterial thrombosis are uncommon in IBD, arterial involvement is even more exceptional. In our case, thrombosis of the ileocolic artery represents a particularly rare and clinically significant finding.

UC during pregnancy poses a clinical challenge for several reasons. Among the most relevant are the unfounded fear of medication teratogenicity, which may lead patients and healthcare providers to discontinue treatment due to inadequate information. Additionally, the tendency to interpret certain pregnancy-related symptoms as “normal” can delay the recognition of disease flares or complications. These factors combined increase the risk of adverse outcomes for both mother and fetus.

To contextualize this case, a narrative review of the literature was conducted using the PubMed database, identifying very few similar reports published to date.

## Case presentation

A 24-year-old woman with a three-year history of poorly managed and uncontrolled UC, currently at 22 weeks of gestation, presented with abdominal pain localized to the mesogastrium radiating to the right iliac fossa for one week, accompanied by general malaise, undocumented fever, and eight episodes of bloody diarrhea per day. On admission, laboratory tests revealed anemia with a hemoglobin level of 8.6 mg/dL, no leukocytosis, and an electrolyte imbalance.

Magnetic resonance imaging (MRI) demonstrated a 9.6 mm appendix, free fluid in the right iliac fossa, and reactive thickening of the cecum. Given the suspicion of acute appendicitis in the setting of a severe ulcerative colitis flare, and recognizing the difficulty in clinically distinguishing the inflammatory signs of both conditions, a diagnostic laparoscopy was performed. Intraoperatively, the appendix was found to be edematous with signs of ischemia, while the ascending colon remained unaffected. An appendectomy was subsequently carried out.

Forty-eight hours postoperatively, the patient developed generalized abdominal pain with signs of peritoneal irritation, fever of 38.5 °C, leukocytosis of 52,000 μL, and a hemoglobin drop to 6.5 mg/dL. Supportive treatment was initiated, and microbiological studies returned positive for *Clostridioides difficile (C. difficile)*. Follow-up MRI revealed free fluid in multiple compartments, thickening of the distal ileum, cecum, and right colon, as well as areas suggestive of microhemorrhages. This allowed ruling out appendicitis as the primary cause of the symptoms and guided the evaluation toward complications related to ulcerative colitis.

**Figure 1:**
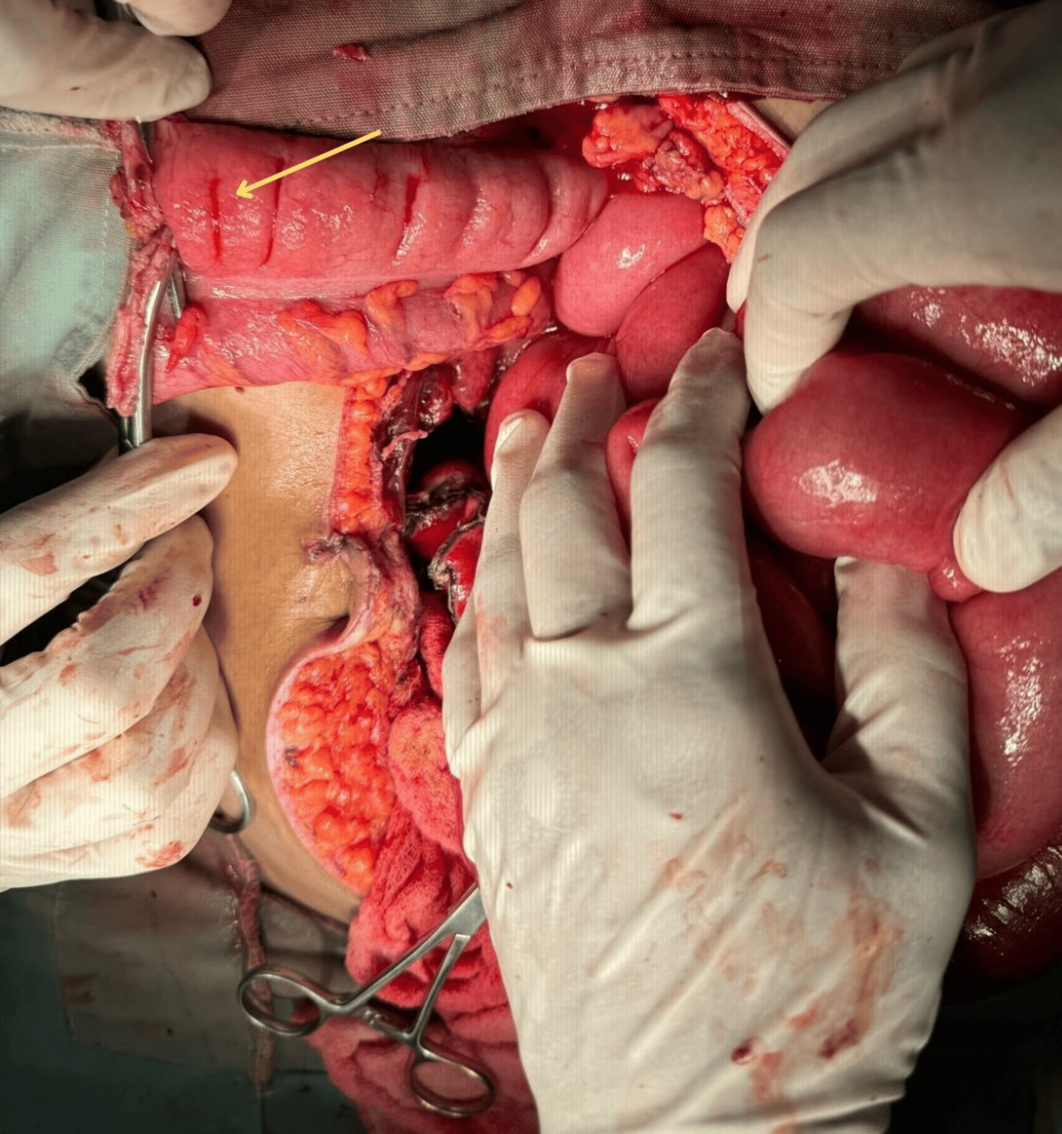
Intraoperative image. The arrow indicates the transverse colon, representing the transition site to healthy tissue. A clear contrast was observed between the ischemic and necrotic segment of the intestine (distal ileum and right colon) and the viable tissue. This visualization confirmed the extent of the arterial lesion and guided the necessary resection.

**Figure 2:**
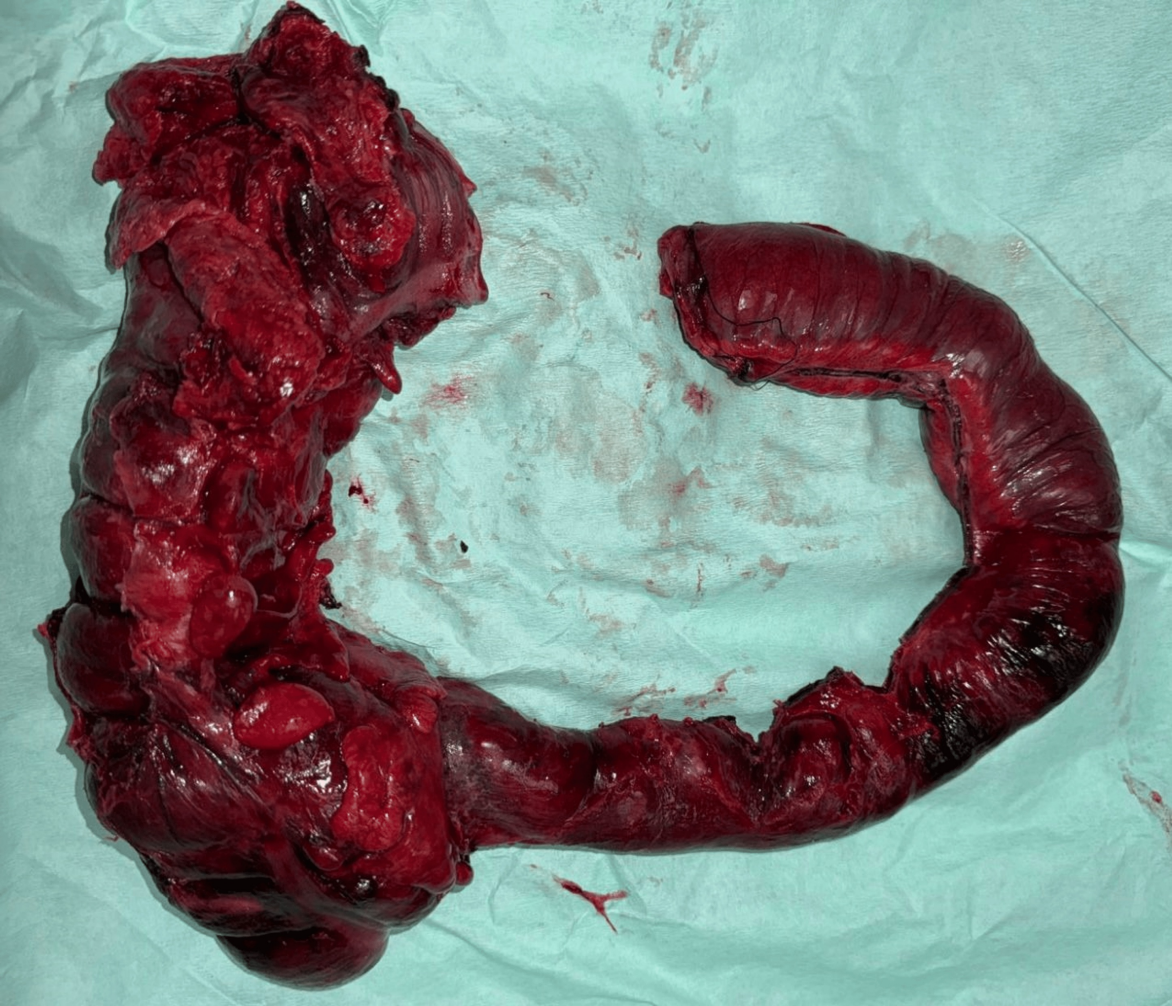
Surgical specimen. Result of partial ileectomy and right colectomy. The specimen showed markedly edematous intestinal tissue, areas of mucosal necrosis, and inadequate tissue perfusion.

In consultation with the gastroenterology, coloproctology, and gyneco-obstetrics departments, an exploratory laparotomy was performed. During the procedure, the transverse colon demonstrated a transition to healthy mucosa (Figure 1), with preserved architecture and no evidence of ischemia or pseudomembranous inflammation. In contrast, the distal ileum and right colon exhibited necrosis, marked edema, and impaired perfusion, which allowed for delineation of the arterial lesion and surgical planning. A partial ileectomy and right colectomy were performed, followed by a Hartmann’s procedure with end ileostomy (Figure 2), revealing areas of transmural necrosis and inadequate tissue perfusion in the resected specimens.

Within the first 12 postoperative hours, absence of fetal cardiac activity was confirmed, prompting labor induction. Histopathological examination confirmed the diagnosis, revealing ileitis and hypoxic-ischemic colitis, with an old thrombus and another undergoing organization in the ileocolic artery, along with vascular congestion. The patient showed a favorable clinical evolution and was discharged on the tenth day of hospitalization.

## Discussion

UC is an IBD of multifactorial origin whose etiology remains unclear, although dietary and environmental risk factors, as well as host factors, genetic susceptibility, and the intestinal microbiota, have been implicated [1-2]. UC represents a clinical challenge during pregnancy due to the delicate balance between disease control and maternal-fetal safety. This case illustrates an unusual but severe complication in a pregnant patient with poorly controlled ulcerative colitis: ileocolic arterial thrombosis resulting in ischemia, colectomy, and fetal loss.

Although there are no statistics available for Latin America, IBD affects approximately 0.7% of the population in Western countries, with diagnosis most frequently made during the reproductive age [3].

Disease activity at conception predicts its course during gestation. It has been shown that patients in remission have up to a 35% risk of relapse during pregnancy, similar to that of nonpregnant women. In contrast, patients who conceive during an active phase of the disease have a risk ranging from 33-79% of ongoing activity or flares during pregnancy and/or the postpartum period [3-4]. While disease activity at conception is an important prognostic factor, in this case, the patient had a three-year history of poor adherence or suboptimal therapeutic management, which likely contributed to the adverse outcome.

The primary clinical manifestation is bloody diarrhea, present in over 90% of cases, frequently accompanied by cramping pain in the lower left quadrant or diffuse colonic pain in patients with pancolitis [1]. However, extraintestinal manifestations can also occur, and the one described in this article is among the least common.

Thromboembolic complications, both venous and arterial, are serious extraintestinal effects of IBD that can worsen disease progression and increase the risk of morbidity and mortality. Although the exact etiology remains unknown, it is believed that chronic inflammation in patients with IBD leads to injury of the intestinal microvasculature. The vascular hypothesis suggests that this endothelial damage plays a significant role in arterial thrombotic complications, especially when associated with a hypercoagulable state [5]. Pregnancy itself represents a physiologically hypercoagulable condition, and when combined with the inflammatory activity of IBD, the risk of thrombotic events increases significantly. In this case, advanced vascular damage was evident, as confirmed by histopathological findings of hypoxic-ischemic colitis.

Several risk factors contribute to the development of thromboembolic events. In a prospective study of pregnant women with IBD, Rottenstreich et al. evaluated thrombin generation and demonstrated that a high body mass index, active IBD, advanced gestational age, and the presence of extraintestinal manifestations were independently associated with a prothrombotic state [6].

Patients with IBD requiring hospitalization have a higher risk of thromboembolic events compared to the non-IBD population. Although thromboembolic complications are not the most frequent manifestations, venous thromboembolic events constitute the majority within this group. In contrast, mesenteric arterial thrombosis in the context of ulcerative colitis is an uncommon cause of acute abdominal pain and is sporadically reported in the literature, leading to limited available evidence [7-8]. Due to its low incidence, the availability of clear guidelines for optimal management of this condition is limited. In this context, the clinical presentation of severe abdominal pain, fever, and signs of peritonitis in a patient with active ulcerative colitis should alert the clinical team to the possible presence of intestinal ischemia, especially when initial studies are inconclusive.

Patients with IBD have an increased risk of developing *C. difficile* infection. Compared to the general population, IBD patients with this infection often differ in several clinical aspects: they tend to be younger, report no recent antibiotic exposure, and more frequently acquire the infection in the community setting [9]. This infection has been shown to worsen IBD outcomes, being associated with higher morbidity and mortality [9-10].

In the case presented, coinfection with *C. difficile* likely acted as a trigger and amplifier of the severe ulcerative colitis flare, promoting a systemic inflammatory state and contributing to the development of the catastrophic arterial thrombotic event. This mechanism has been described in the literature, where active inflammation and microorganism-induced dysbiosis are linked to coagulation disturbances and an increased risk of thromboembolic events. Timely identification and treatment of this infection are therefore essential to prevent adverse outcomes in patients with IBD, particularly during pregnancy, when therapeutic management is more complex and clinical decisions must balance maternal and fetal risks.

Mesalamine has been established as the first-line treatment for patients with mild-to-moderate ulcerative colitis, as it has been shown to reduce levels of Regulated upon Activation, Normal T cell Expressed and Secreted (RANTES), a proinflammatory chemokine elevated in patients with IBD and associated with platelet dysfunction. In addition, it inhibits platelet activation, which may reduce the risk of thromboembolism [11]. With the exception of methotrexate, agents such as 5-aminosalicylates, corticosteroids, thiopurines, and anti-tumor necrosis factor (TNF) agents are considered safe during pregnancy and breastfeeding [12].

Surgical management of IBD during pregnancy follows the same principles as in nonpregnant patients. When the disease is active and unresponsive to medical therapy, the risk to the fetus is greater if the condition persists than if surgery is performed. Surgical decision-making during pregnancy is always complex, but in cases of refractory active disease and complications such as intestinal ischemia, surgery is indicated and may, in fact, improve maternal outcomes. Although surgery can be safely performed in any trimester, some small studies have reported a potential increase in the risk of spontaneous miscarriage [4].

A literature search was conducted in PubMed using the terms ‘case report,’ ‘pregnancy,’ ‘ulcerative colitis,’ and ‘thrombosis,’ covering the last 30 years. Only two cases related to thrombosis were identified (Table 1), and no articles reported arterial thrombosis in the gastrointestinal tract.

**Table 1:** Reported cases of thrombosis due to UC in the context of pregnancy.

Author	Age	Gestational Age	Thrombotic Event	Clinical Presentation	Treatment	Outcome
Akhoundizardini et al. [13]	35	32 weeks / postoperative puerperium	Cavernous sinus thrombosis & pulmonary embolism	Cough, mild dyspnea, headache, fever, myalgia, tachycardia	Anticoagulation (Warfarin, Heparin)	Complete recovery; discharged
Bhandari et al. [14]	29	30 weeks / physiological puerperium	Left ovarian vein thrombosis	Abdominal pain	Heparin	Complete recovery; discharged

The first case involved a 35-year-old woman with a 15-year history of UC who experienced a disease flare during pregnancy. The pregnancy resulted in delivery at 32 weeks of gestation due to intrauterine growth restriction. Two months postpartum, the patient developed progressive headache, myalgia, fever, and respiratory difficulty. Imaging studies confirmed cavernous sinus thrombosis and pulmonary embolism, and she was treated with anticoagulants [13].

The second case involved a 29-year-old woman with a history of UC who experienced a flare at 22 weeks of gestation. At 30 weeks, she had a preterm delivery, and during the immediate postpartum period, she developed abdominal pain. Imaging studies revealed thrombosis of the left ovarian vein, which was treated with heparin [14].

The scarcity of reported cases highlights the extreme rarity of vascular thrombosis associated with ulcerative colitis during pregnancy. The available literature predominantly describes venous thrombosis, mainly in pelvic or systemic contexts. In contrast, our case involves arterial thrombosis, a significantly less common presentation, underscoring the uniqueness and clinical relevance of this report.

Unfortunately, the severity of the inflammatory process and systemic imbalance resulted in fetal loss just hours after the surgical procedure. The course of this case reflects the diagnostic complexity in patients with active ulcerative colitis during pregnancy, where the overlap of gastrointestinal symptoms with physiological gestational changes can delay the recognition of severe complications. Persistent abdominal pain despite the initial appendectomy, along with laboratory findings indicating extreme leukocytosis and a drop in hemoglobin, prompted reassessment toward a rare vascular complication. Histopathological confirmation of ileocolic arterial thrombosis, an exceptional finding in patients with IBD, given that most thromboembolic events in this population are venous-provides a unique context and highlights the need for a high index of suspicion in cases of unexpected clinical deterioration. Despite the adverse outcome, surgical intervention allowed for control of the ischemic lesion and prevented additional complications. This scenario emphasizes the importance of strict multidisciplinary follow-up in pregnant women with active IBD, as well as the necessity for early and accurate diagnostic strategies to identify rare but potentially catastrophic complications.

## Conclusions

Given the link between acute flares of IBD and thrombotic events, maintaining a high index of suspicion is essential for the early diagnosis of rare complications, such as arterial thrombosis, and for providing timely treatment. In pregnant patients, close medical monitoring is crucial to prevent disease reactivations that could jeopardize both maternal and fetal health.

This particular case highlights the rarity of ileocolic arterial thrombosis as a complication of active ulcerative colitis, an extremely uncommon event. Identification of this complication required clinical reassessment in the face of persistent symptoms following the initial appendectomy, underscoring the difficulty of recognizing ischemic signs superimposed on inflammatory activity and the physiological changes of pregnancy.

The case emphasizes the importance of a multidisciplinary approach and the need to raise awareness about the risks associated with discontinuation of medical therapy due to concerns about the teratogenicity of first-line drugs. Furthermore, it highlights the relevance of ensuring stable disease remission at conception and throughout pregnancy, as well as the importance of considering unusual vascular complications early when the clinical course does not align with the expected patterns of IBD.
